# Regulatory challenges of personalized medicine in the real world: are the right patients being treated with CD19-targeting CAR T-cells?

**DOI:** 10.1038/s44321-026-00436-2

**Published:** 2026-04-27

**Authors:** Attila Sebe, Rafael Hernani, Jan Müller-Berghaus

**Affiliations:** 1 Paul-Ehrlich-Institut, Langen, Germany; 2 Hematology Department, Hospital Clínico Universitario INCLIVA Biomedical Research Institute, Valencia, Spain

**Keywords:** Cancer, Immunology

## Abstract

Despite the clinical success of CD19-directed CAR T-cell therapies, less than 50% of patients achieve long-term remission. Emerging evidence indicates that loss or reduced expression of CD19—due to mutations, deletions, alternative splicing—is a significant, underrecognized mechanism of treatment failure. Notably, CD19-negative subpopulations can be detected even prior to therapy, and patients with low CD19 expression consistently show poorer outcomes. Although current clinical guidelines do not mandate routine CD19 testing before treatment, this situation reflects both scientific and technical challenges. Importantly, patients with partial/ low CD19 expression may still benefit from therapy, complicating the definition of “target positivity.” From a regulatory perspective, targeted therapies should ideally be used only when target expression is confirmed. This principle is difficult to implement, considering the scarcity of data and the difficulties of current diagnostic tools, risking both undertreatment and overtreatment. EU regulators have addressed the issue by introducing harmonized warnings in product information, but more is needed. We highlight this regulatory-clinical gap and advocate for improved diagnostic standards, better data integration, and dialogue among clinicians, developers, and regulators.

## Abstract (web summary)

This Perspective by A. Sebe, R. Hernani and J. Müller-Berghaus discusses how low or variable CD19 expression may affect the effectiveness of CD19-targeted therapies and calls for improved diagnostics, data integration, and regulatory alignment to improve patient outcomes.

## Graphical abstract

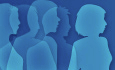

CD19 is a type I transmembrane glycoprotein and a member of the immunoglobulin superfamily. The CD19 gene, located on the short arm of chromosome 16 (16p11.2), contains 15 exons and encodes the CD19 molecule, which consists of 556 amino acids. Structurally, CD19 features an extracellular domain composed of two tandem immunoglobulin-like loops responsible for ligand binding and interaction with other co-receptors, a transmembrane region, and a cytoplasmic domain containing multiple conserved tyrosine residues. These residues serve as potential docking sites for downstream signaling molecules such as Src-family kinases (e.g., Lyn) and phosphoinositide 3-kinase (PI3K) (Wang et al, 2012).

CD19 expression is restricted exclusively to B cells, beginning at the early pro-B-cell stage and continuing through mature B cells, but it is lost upon differentiation into plasma cells. It functions as a central component of the B cell co-receptor complex, which also includes CD21 (complement receptor 2), CD81 (a tetraspanin), and CD225. The association of these molecules enables CD19 to modulate B-cell receptor (BCR) signaling with high sensitivity (Tedder et al, 1997). Upon antigen binding to the BCR, CD19 is phosphorylated by Src-family kinases, leading to the recruitment of signaling enzymes. This lowers the activation threshold, enhances proliferation and differentiation, and helps regulate B-cell tolerance (Fruman and Bismuth, 2009; Dal Porto et al, 2004). These functions make CD19 a key regulator of normal B-cell development, immune response, and homeostasis.

CD19 expression is tightly linked to B-cell lineage and is retained on most B-cell malignancies, including B-cell acute lymphoblastic leukemia (B-ALL), chronic lymphocytic leukemia, and various non-Hodgkin lymphomas, such as follicular lymphoma, primary mediastinal lymphoma, and diffuse large B-cell lymphoma (DLBCL). Aberrant expression or dysregulation of CD19-associated signaling pathways can contribute to malignant transformation and disease progression by promoting uncontrolled proliferation and survival of malignant B cells (Wang et al, 2012). Clinically, CD19 serves as a valuable biomarker for diagnosis in B-cell cancers. Its consistent expression on malignant B cells has also made it a prime target for therapeutic intervention. Accordingly, CD19 is the target of several authorized medicinal products such as bispecific T-cell engagers (blinatumomab), antibody-drug conjugates (loncastuximab tesirine), monoclonal antibodies (tafasitamab), and CAR T-cell therapies.

Currently, there are five CD19-targeting CAR T-cell therapies with a marketing authorization in the EU: tisagenlecleucel, axicabtagene-ciloleucel (axi-cel), lisocabtagene-maraleucel, brexucabtagene autoleucel, and obecabtagene autoleucel. These therapies are currently approved for hematological malignancies of B-cell origin, including DLBCL and B-ALL. As CD19 is widely considered to be a stably expressed antigen in these malignancies (Whilding and Maher, 2015; Maude et al, 2015), CD19 expression was generally not an entry criterion for inclusion in the pivotal clinical trials of these CAR T cells.

Despite remarkable advances in treating some hematological malignancies, it is important to note that only about 50% or fewer patients achieve long-term benefits (in the sense of long-term remission without evidence of disease) following CD19-targeted CAR T-cell therapies, regardless of indication. In the ZUMA-1 trial, patients treated with axi-cel achieved a 5-year progression-free survival (PFS) rate of 31.8% (95% CI, 22.9–41.1) and the 5-year overall survival (OS) rate of 42.6% (95% CI, 32.8–51.9), while a meta-analysis of CAR T-cell treated patients with relapsed/refractory B-ALL revealed 5-year OS rate of 44.1% (95% CI, 36.3–53.5). Some patients are refractory, while ~40–50% relapse after treatment (Chong et al, 2021; Neelapu et al, 2023; Elsallab et al, 2023). Understanding the factors that contribute to these outcomes is crucial for optimizing these treatments. Here, we will discuss the evolving role and understanding of CD19 expression as a potential limiting factor contributing to these outcomes. We summarize current knowledge on CD19 expression patterns in hematologic malignancies and explore their relationship with patient outcomes. Although low or absent CD19 expression has not been a primary focus in the field, accumulating evidence highlights its clinical relevance. Therefore, a broader discussion is warranted, and future developments should consider these findings. We propose that the clinical community plays an important role in refining the most appropriate use of these therapies. Additionally, we discuss key considerations that may influence regulatory approaches and patient selection criteria.

CD19 is one of three B-lineage markers—CD19, CD79a, and cCD22—used in the diagnostic workup of ALL, where positivity for at least two of these markers is sufficient for establishing B-lineage disease. This immunophenotype is assessed by flow cytometry, typically performed on bone marrow aspirates obtained as part of standard diagnostic procedures (Hoelzer et al, 2016). In contrast, clinical guidelines did not require CD19 testing prior to CD19-directed CAR T-cell therapy in relapsed or refractory lymphoma, except in certain EU countries (e.g., Germany), where confirmation of CD19 expression is a prerequisite for reimbursement in patients previously treated with other CD19-targeted therapies (Medical Service). The NCCN Guidelines only indicate CD19 testing by flow cytometry for the first diagnosis of DLBCL (NCCN Clinical Practice Guidelines in Oncology (NCCN Guidelines)). According to the earlier version of the DLBCL diagnosis guideline of the European Society of Medical Oncology (ESMO) a morphological diagnosis of DLBCL should be confirmed by immunophenotypic investigations—using immunohistochemistry, flow cytometry, or a combination of both—although standard immunohistochemical panels for DLBCL typically do not include CD19 (Tilly et al, 2015). Yet the most recent, 2025 version of the lymphoma guideline of the ESMO proposes a rebiopsy at each DLBCL relapse to evaluate the immunophenotype, including CD19 by immunohistochemistry or flow cytometry (depending on the type of patient material), considering the evolving therapeutic landscape in this setting (Eyre et al, 2025). Accurate assessment of CD19 expression in lymphoma is therefore increasingly important, despite the limitations inherent to current diagnostic methods. CD19 staining by immunohistochemistry (IHC) is not standardized and is subject to potential biases related to subjectivity and fixation artifacts. Given that various CD19 antibodies targeting different epitopes are available, and most laboratories use only a single CD19 antibody for IHC, false-negative results are possible (Bailly et al, 2022). Quantitative FCM would likely be the ideal diagnostic method for determining CD19 expression in tumor cells. However, in lymphoma cases, FCM analysis is much more challenging: lymph node samples often do not arrive fresh for FCM, and even when they do, cellularity may be insufficient for cytometric labeling (Berger et al, 2022).

CD19-negative newly diagnosed DLBCL and B-ALL cases have been reported in the literature, with incidences up to 15% in certain patient cohorts as assessed by IHC and flow cytometry (Masir et al, 2006; Kimura et al, 2007; Ghodke et al, 2017; Delage et al, 2019). Notably, single-cell RNA sequencing has identified CD19-negative subclones in a B-ALL patient prior to CAR T-cell therapy, following a relapse after chemotherapy (Rabilloud et al, 2021). While differences in assay sensitivity—particularly in IHC—may partly explain these findings, the detection of CD19-negative populations by both FCM and single-cell transcriptomics supports the clinical relevance of CD19-negative disease. Loss of CD19 expression has emerged as one of the key mechanisms of relapse following CD19-directed CAR T-cell therapy (Lemoine et al, 2021; Ruella and Maus, 2016; Plaks et al, 2021). In relapsed B-ALL, several mechanisms of CD19 antigen escape have been described, including hemizygous deletions involving the CD19 locus, de novo frameshift and missense mutations in exon 2, and the generation of alternatively spliced CD19 transcripts (Sotillo et al, 2015). Importantly, similar alterations have also been detected at diagnosis—prior to any CD19-targeted therapy—and even in individuals without leukemia (Fischer et al, 2017). These changes can lead to loss of the CAR-binding epitope. For example, frameshift mutations may introduce premature stop codons, resulting in truncated CD19 proteins lacking the epitope encoded by exon 4. Importantly, the epitope encoded by exon 4 is targeted by the commonly used FMC63-based CAR construct (Sommermeyer et al, 2017).

Axi-cel was the first CAR T-cell therapy to receive regulatory approval for LBCL, based on data from the single-arm, uncontrolled ZUMA-1 trial (Neelapu et al, 2017). All patients enrolled in ZUMA-1 had histologically confirmed large B-cell lymphoma (LBCL), but CD19 expression was not an inclusion criterion. Although CD19 IHC data were collected, among the nine patients with negative CD19 IHC staining, five achieved a clinical response. During the marketing authorization review, CD19 negativity was closely examined. The negative IHC results were primarily attributed to potential false negatives caused by antigen degradation over time or the limited sensitivity of IHC in detecting CD19 in heterogeneous tumor populations. It was concluded that tumor cells classified as CD19-negative by IHC might still express CD19 at levels sufficient to trigger CAR T-cell activation and cytotoxicity (Yescarta European Public Assessment Report (EPAR)). Notably, CD19-negative relapse occurred in approximately 30% of patients treated with axi-cel in ZUMA-1, possibly due to treatment-related selection of tumor cells with markedly reduced CD19 protein expression (Plaks et al, 2021).

CD19 expression again became a focus during the evaluation of axi-cel for its second-line indication. Data were obtained in the ZUMA-7 trial, which randomized patients to axi-cel or standard of care (chemoimmunotherapy and autologous stem cell transplantation) (Locke et al, 2022). Patients with histologically confirmed LBCL were enrolled without requiring CD19 expression for trial enrollment. Among the axi-cel-treated group, 13 patients were CD19-negative by IHC, compared to 12 in the standard care arm. Subgroup analysis revealed that CD19-negative patients in the axi-cel arm were less likely to achieve a complete response than CD19-positive patients (38% vs. 67%). Moreover, CD19-negative patients did not show better complete response rates than those receiving standard care (38% vs. 42%) (Yescarta European Public Assessment Report (EPAR)). Differences in outcome were seen regardless of whether CD19 expression was measured by IHC or mRNA levels. When patients were stratified by median expression values, event-free survival (EFS) was better in axi-cel-treated patients with higher-than-median CD19 gene and protein expression compared to those with lower (≤ median) expression. However, this approach is limited because it compares only two groups, defined solely by the median. A more granular analysis using three or four expression-based subgroups compared to standard of care would likely have more clearly demonstrated the poorer outcomes in patients with lower CD19 expression. (Locke et al, 2024). Similar observations have been made across products and indications: in patients treated with tisagenlecleucel, for example, CD19-dim B-ALL cases showed a trend toward a higher rate of failure to achieve deep remission (Pillai et al, 2019). However, median CD19 expression is likely an insufficient and less relevant metric for predicting individual patient outcomes, considering the lack of validated cut-off values for CD19 expression. Pretreatment low surface CD19 density is associated with progressive disease following axi-cel treatment: the risk of relapse increases in LBCLs expressing a median of ≤3000 CD19 molecules per cell before therapy, as measured by quantitative FCM. Quantitative FCM, rather than semiquantitative IHC, may better identify LBCL patients a priori at risk of relapse after axi-cel treatment (Spiegel et al, 2021). No such analysis incorporating this threshold has been reported for ZUMA-7 cohorts.

The importance of CD19 antigen density was also addressed in experiments comparing two CD19 CAR designs that differ in their costimulatory domains: a 4-1BBζ-based construct and a CD28ζ-based construct. These CAR T cells were tested against NALM6 clones engineered to express varying levels of surface CD19. The 4-1BBζ CAR showed reduced cytotoxicity, proliferation, and cytokine production against low CD19 targets, whereas the CD28ζ CAR maintained killing and proliferative capacity even at low antigen densities. Across all CD19 levels, the CD28ζ CAR T cells produced more IL-2, and at low densities, only the CD28ζ construct generated measurable IL-2 (Majzner et al, 2020).

A recent study proposed a novel approach to address these uncertainties. Based on their findings, the authors suggested routinely performing qPCR on all pre-CAR-T biopsies where CD19 IHC is negative. In a retrospective study of 51 adult LBCL patients treated with commercial axi-cel, 35 were CD19-positive by IHC, while 16 were IHC-negative. Among the 16 initially CD19-negative patients, qPCR reclassified six as CD19-positive (IHC−qPCR + ), while the remaining 10 were confirmed as truly negative (IHC−qPCR − ). This indicates a 20% prevalence of true CD19-negative patients within this cohort. As expected, the CD19-negative group showed poorer 1-year progression-free survival and a trend toward shorter duration of response. Notably, only one CD19-negative patient remained alive and disease-free at last follow-up (6 months), having previously responded to bridging therapy (Hernani et al, 2025). Quantitative polymerase chain reaction (qPCR) may be as sensitive as FCM, and gene expression patterns correlated well with the protein phenotype, with the primary advantage that mRNA can be easily extracted from paraffin-embedded tissues, avoiding the need for fresh samples (Sandstedt et al, 2015).

These data make it clear that not all patients benefit from CD19-directed CAR T-cell therapies. While CD19-negative B-cell subpopulations may be present prior to CAR T-cell treatment, this should not automatically exclude patients from CAR T-cell therapy. The presence of such CD19-negative clones likely identifies a patient population at higher risk for CD19-negative escape, necessitating closer monitoring. However, true loss of CD19 expression—due to mutations, deletions, or alternative splicing—leads to therapy failure. Beyond complete loss, low CD19 expression requires a better understanding to determine the threshold necessary for CAR T-cell activation. As discussed above, studies show that low or absent CD19 expression correlates with poorer clinical outcomes in patients treated with CD19-targeting CAR T cells (Spiegel et al, 2021; Locke et al, 2024). Data from axi-cel and tisagenlecleucel suggest this is a general issue for all CD19-directed CAR T-cell therapies. Although some CD19-negative patients exhibit transient responses, which may be influenced by bridging or lymphodepleting chemotherapy, the overall efficacy in this group remains limited.

Significant technical and methodological challenges remain in accurately defining CD19 positivity and negativity, underscoring the need for improved quantification methods. From both a scientific and regulatory standpoint, it could be generally postulated that targeted therapies should ideally be administered only when the target is present. However, this principle entails risks due to current diagnostic limitations and uncertainties. On one hand, there is the potential risk of treating target-negative patients, which could delay access to alternative therapeutic options—a non-negligible concern in the evolving landscape of CAR T-cell therapies, as they are increasingly being used in earlier lines of treatment. Conversely, excluding borderline or low-expressing target-positive patients risks denying potentially effective treatment.

The question of CD19 expression thresholds is an increasingly important consideration in the context of sequencing different CD19-targeting agents. However, direct comparisons of the target expression levels required for efficient tumor cell killing are of limited value, given the fundamental differences in their mechanisms of action. CAR T-cell function is independent of natural signaling and interactions with other immune cells. In contrast, the function of antibody-based therapies is dependent on and can be limited by factors beyond the characteristics of the CD19-expressing target cell. For instance, monoclonal antibodies like tafasitamab require the triggering of immune effector functions, such as antibody-dependent cellular cytotoxicity, as well as an active host immune system. The activity of T-cell engagers (e.g., blinatumomab) is influenced by the specific affinities of both the CD19 and CD3 binding domains, and is heavily dependent on the functional integrity of the engaged T cells. Lastly, antibody-drug conjugates like loncastuximab tesirine combine an anti-CD19 antibody with a cytotoxic payload, and their efficacy relies not only on CD19 binding but also on the internalization and intracellular action of the payload. Furthermore, in a context of heterogenous target expression within the tumor, use of cell-permeable payloads released from the ADC exploits the mechanism of “bystander killing”, enabling even eradication of target-low to -negative tumor cells. Therefore, the recent recommendation in the ESMO Lymphoma Guideline (Eyre et al, 2025) to assess CD19 expression, at least in the relapse setting, is welcome.

There is a clear need for ongoing dialogue among CAR T-cell therapy developers, clinicians, and regulators to address the challenges outlined here. Regulatory measures have been mostly exhausted by introducing specific, harmonized warnings in the labeling of all CD19-targeting CAR T cells approved in the EU. Given this, it is crucial that professional societies and clinical networks continue to advance this work. Moreover, treatment guidelines must keep pace with rapid scientific advances, ensuring they align with and reflect the latest scientific understanding in the field. New and more precise methods for detecting CD19 expression should be thoroughly explored to better identify the patients who will benefit most from these targeted therapies. Collecting and analyzing expression data alongside clinical outcomes—such as treatment response—will be essential both in clinical practice and prospective clinical trials. To further strengthen the evidence base, systematic additional data collection should be encouraged. The latest ESMO Lymphoma Guideline recommends assessment of CD19 expression at least in the relapse setting (Eyre et al, 2025). Incorporating this parameter into post-approval registry data collection could facilitate the establishment of a structured evidence base to inform future scientific, clinical, and regulatory decision-making. In addition, novel designs may further improve therapeutic outcomes in CD19-low or CD19-negative tumors: CD20/CD19 tandem CAR T cells have shown promising clinical results (Balke-Want et al, 2026; Shah et al, 2024), and the proposed HIT receptors appear to be at least tenfold more sensitive to antigen than conventional CARs (Mansilla-Soto et al, 2022).

Ongoing research is vital to fully evaluate the true clinical benefits of CD19-directed CAR T-cell therapies and to confirm that their prominent role in treatment is supported by strong clinical evidence. Only through these combined efforts can the promise of CAR T-cell therapies be fully realized, ensuring their continued relevance and effectiveness as their use expands in clinical practice.

## Supplementary information

Peer Review File
