# Supplementary material for: Regulatory challenges of personalized medicine in the real world: are the right patients being treated with CD19-targeting CAR T-cells?
Source: EMBO Mol Med. 2026 Apr 27;18(6):2007–11. doi: 10.1038/s44321-026-00436-2 (PMC13270044; doi:10.1038/s44321-026-00436-2)
Supplement: Supplementary file 1 — Peer Review File [file 44321_2026_436_MOESM1_ESM.pdf]

EMBO Molecular Medicine

# Are the Right Patients Being Treated with CD19-Targeting CAR T-Cells?

Attila Sebe, Rafael Hernani, and Jan Müller-Berghaus

*Corresponding author: Attila Sebe (Attila.Sebe@pei.de)*

**Review Timeline:**

| | |
|---|---|
| Submission Date: | 4th Dec 25 |
| Editorial Decision: | 4th Feb 26 |
| Revision Received: | 23rd Mar 26 |
| Editorial Decision: | 14th Apr 26 |
| Revision Received: | 20th Apr 26 |
| Accepted: | 21st Apr 26 |

*Editor: Lise Roth*

## Transaction Report:

(Note: With the exception of the correction of typographical or spelling errors that could be a source of ambiguity, letters and reports are not edited. Depending on transfer agreements, referee reports obtained elsewhere may or may not be included in this compilation. Referee reports are anonymous unless the Referee chooses to sign their reports.)

4th Feb 2026

Dear Dr. Sebe,

Thank you for the submission of your Perspective to EMBO Molecular Medicine. Please accept my apologies for the delay in responding, which is due to our office's reduced activity over the holiday season and the high volume of manuscripts we have received recently. We have now received feedback from the experts who agreed to evaluate your manuscript.
As you will see from the reports below, they both found the article interesting, important and timely. They nevertheless make a few suggestions to improve the interest and impact of your work.
We would therefore welcome a revised version of your manuscript that would address these points.

Please attach a covering letter giving details of the way in which you have handled each of the points raised by the referees.

Looking forward to receiving your revised manuscript at your earliest convenience.

With kind regards,

Lise Roth

Lise Roth, Ph.D
Senior Editor
EMBO Molecular Medicine

When submitting your revised manuscript, please include:

1) a .doc formatted version of the manuscript text (including Figure legends and tables)
2) Separate figure files
3) a letter INCLUDING the reviewer's reports and your detailed responses to their comments.

Also, and to save some time should your paper be accepted, please read below for additional information regarding some features of our research articles:

4) Disclosure and competing interest statement: Please include a statement declaring any competing commercial interests in relation to your submitted work.

5) Please note that we now mandate that all corresponding authors list an ORCID digital identifier. This takes <90 seconds to complete. We encourage all authors to supply an ORCID identifier, which will be linked to their name for unambiguous name identification.

Currently, our records indicate that the ORCID for your account is 0000-0001-7814-5144.

Please click the link below to modify this ORCID:
Link Not Available

***** Reviewer's comments *****

Referee #1 (Remarks for Author):

This is an interesting and balanced perspective article that discusses the issue of CD19 expression on tumor cells affecting the clinical outcome of CD19 CAR T and presumably other CD19 targeted therapies. The discussion is relevant given the multiple

approvals, high cost, and toxicities of CD19 targeted therapies making it important to ensure that patients with B cell malignancies who are treated will most likely to derive maximal benefit. There are a few comments that I feel would improve the manuscript.

Comments.
1. The authors review published work that describes CD19 negative escape and the findings that mutations, splicing alterations, and low CD19 expression prior to therapy can affect key clinical outcomes such as PFS and OS. There are vagaries in much of this retrospective data, which is relying on subgroup analysis with less than optimal measurements of CD19. While this is discussed, I would encourage more clarity on the author's views. Could the authors perhaps suggest a path forward here -- perhaps a post approval recommendation by regulatory agencies to collect data on CD19 expression, even if this is done with readily available assays such as IHC?

2. The authors discuss the challenges of the ideal, which would be quantitative measurements of CD19 that could ideally be performed on single cells given that tumors - even when originating in a single lineage -- are notoriously genetically and phenotypically heterogeneous. Firm recommendations on alternative measurements of CD19 that could be maximally useful are not provided apart from noting potential advantages of a qPCR assay to detect mRNA transcript expression that could be used on FFPE samples. A comment on the feasibility of this idea given that patients often require urgent therapy. For example, if a patient is referred to a treatment center, it would require obtaining diagnostic blocks, extracting RNA and performing the qPCR in a clinical lab with an assay that has been validated with defined cut-offs. I am not sure how practical this is unless given the limited alternative therapies for refractory B cell malignancies.

3. It would be ideal for completeness to briefly discuss advances in the field that may address the issue of CD19 negative or low tumor variants, and that may reduce the need for measuring CD19. For example bispecific CAR T cells are in advanced clinical trials, including randomized studies comparing commercial CD19 CAR T cells: and more sensitive receptor designs (HIT, STAR, chimeric TCR) that can better target antigen low tumor cells and have been published. In the case of the chimeric TCR, targeting 2 antigens (eg CD19, CD22) can be accomplished with higher sensitivity.

Referee #2 (Remarks for Author):

Sebe et al have addressed a very important topic. Relapse after CD19-targeted CAR-T-cells is observed routinely, and the question is, if detection of CD19 expression should be a condition for treatment.

The manuscript is well conceived, but some thoughts could be further expanded:

- Relapse to CD19 CART cells can also present with CD19 positivity. Antigen negativity or loss is not the only mechanism of relapse.
- In case a patient shows CD19 expression, but not homogeneously in all cells, or harbors „CD19 negative clones". It is not at all clear, if these „clones" or cells will be able to reinitiate relapse, or not. In fact, in case any of these patients are cured by CD19 CART-cells only, it would argue that these CD19 positive cells are not able to reinitiate disease or can be cleared differently.
- Patients with leukemia/lymphoma often have disease at different sites oft he body. Is it possible that CD19 expression varies between sites? How shall we investigate this - it is not possible to analyze all.
- Low expression: might be absolutely sufficient for CART cells to recognize.
- Negative expression: can be false negative depending on the antigen used. This could be even more highlighted.
- Negative expression: I agree that in such a case at least dual CARs (CD19+ another target) would be much better.

Target expression is a very big topic for CAR-T cell development, but also for antibody development. Please also make a comparison to what antigen expression is required for antibody treatments.

Overall, this is a very important manuscript.-

Our answers to the questions raised by Reviewer 1:

**1. The authors review published work that describes CD19 negative escape and the findings that mutations, splicing alterations, and low CD19 expression prior to therapy can affect key clinical outcomes such as PFS and OS. There are vagaries in much of this retrospective data, which is relying on subgroup analysis with less than optimal measurements of CD19. While this is discussed, I would encourage more clarity on the author's views. Could the authors perhaps suggest a path forward here -- perhaps a post approval recommendation by regulatory agencies to collect data on CD19 expression, even if this is done with readily available assays such as IHC?**

We thank the reviewer for this insightful comment. We fully agree that the inherent limitations of retrospective data — including non-standardized assays and small patient cohorts — restrict the ability to draw firm conclusions. We would also like to clarify that, from a regulatory perspective, the available instruments to address this issue have been exhausted at this stage.

One of the key objectives of this perspective article is to raise awareness of this important gap. The formulation of regulatory recommendations would require relevant clinical guidelines, which are currently lacking. Although the recent update of the ESMO Lymphoma Guideline (August 2025) represents an important first step — as already briefly mentioned in the manuscript — comprehensive guidance is not yet available.

We agree that the reviewer’s suggestion regarding post-approval data collection is highly relevant and warrants discussion within the framework of this manuscript. In response to the request for a clearer “path forward,” we have revised the manuscript to explicitly advocate for systematic additional data collection, in line with the reviewer’s proposal (lines 233-237).

Specifically, based on the latest ESMO Lymphoma Guideline, CD19 assessment is recommended at least in the relapse setting. Incorporating this parameter into post-approval registry data collection could establish a structured evidence base to inform future regulatory and scientific decision-making, together with upcoming scientific publications and a changing guideline landscape.

**2. The authors discuss the challenges of the ideal, which would be quantitative measurements of CD19 that could ideally be performed on single cells given that tumors - even when originating in a single lineage -- are notoriously genetically and phenotypically heterogeneous. Firm recommendations on alternative measurements of CD19 that could be maximally useful are not provided apart from noting potential advantages of a qPCR assay to detect mRNA transcript expression that could be used on FFPE samples. A comment on the feasibility of this idea given that patients often require urgent therapy. For example, if a patient is referred to a treatment center, it would require obtaining diagnostic blocks, extracting RNA and performing the qPCR in a clinical lab with an assay that has been validated with defined cut-offs. I am not sure how practical this is unless given the limited alternative therapies for refractory B cell malignancies.**

The reviewer raises a valid point. However, as outlined in our response to Question 1, it is currently challenging to provide a definitive position on this aspect. From a regulatory perspective, this is primarily a matter for the relevant learned societies to assess and to develop feasible, consensus-based solutions. Any subsequent regulatory action would need to be based on such clinical guidance, should it become available.

**3. It would be ideal for completeness to briefly discuss advances in the field that may address the**

**issue of CD19 negative or low tumor variants, and that may reduce the need for measuring CD19. For example bispecific CAR T cells are in advanced clinical trials, including randomized studies comparing commercial CD19 CAR T cells: and more sensitive receptor designs (HIT, STAR, chimeric TCR) that can better target antigen low tumor cells and have been published. In the case of the chimeric TCR, targeting 2 antigens (eg CD19, CD22) can be accomplished with higher sensitivity.**

We thank the reviewer for this valuable comment. In response, we have added a dedicated sentence outlining the potential improvements that may be achieved with the introduction of novel CAR T-cell constructs into clinical practice (lines 237-241).

<u>Our answers to the questions raised by Reviewer 2:</u>

**- Relapse to CD19 CART cells can also present with CD19 positivity. Antigen negativity or loss is not the only mechanism of relapse.**

We thank the reviewer for this important comment. Although it was our intention to reflect this aspect in the original text, we have now revised the manuscript to clarify explicitly that low or absent CD19 expression represents only one of several potential mechanisms underlying relapse (line 125).

**- In case a patient shows CD19 expression, but not homogeneously in all cells, or harbors „CD19 negative clones". It is not at all clear, if these „clones" or cells will be able to reinitiate relapse, or not. In fact, in case any of these patients are cured by CD19 CART-cells only, it would argue that these CD19 positive cells are not able to reinitiate disease or can be cleared differently.**

The reviewer raises a fundamental point regarding clonal heterogeneity: the presence of CD19-negative cells does not inherently guarantee relapse if those cells lack the capacity for self-renewal or 'stemness.'
We agree that patients with heterogeneous expression could achieve long-term remission
However, from a clinical risk-stratification perspective, the presence of CD19-negative or 'low' clones represents a significant selection pressure. In many clinical cohorts, these pre-existing sub-clones are the primary drivers of lineage-switch or marker-escape relapses.
We have updated the manuscript to reflect this nuance, noting that while heterogeneous expression does not strictly preclude a cure, it likely identifies a patient population at higher risk for CD19-negative escape, necessitating closer monitoring (lines 201-203).

**- Patients with leukemia/lymphoma often have disease at different sites oft he body. Is it possible that CD19 expression varies between sites? How shall we investigate this - it is not possible to analyze all.**

The reviewer correctly identifies a critical hurdle in personalized immunotherapy: spatial heterogeneity. It is indeed possible—and documented—that CD19 expression levels vary across different disease sites within the same patient, influenced by local selective pressures or clonal divergence.
While we agree that analyzing every site via biopsy is impossible, the lack of clinical guidelines in this respect represent a further gap that may need to be addressed in the future.

**- Low expression: might be absolutely sufficient for CART cells to recognize.**

As we cite the paper by Spiegel et al., it is important to emphasize that there are likely clinically relevant thresholds that may limit antigen recognition and influence clinical outcomes. The practical

applicability and clinical translation of these findings should be carefully evaluated by clinicians and the relevant learned societies.

**- Negative expression: can be false negative depending on the antigen used. This could be even more highlighted.**

We have addressed the possibility of false-negative results at two points in the manuscript (lines 112, 142), with reference to a relevant publication (Bailly et al). Expanding further on this aspect would, in our view, risk diluting the focus and impact of our key observations and conclusions.

**- Negative expression: I agree that in such a case at least dual CARs (CD19+ another target) would be much better.**

We thank the reviewer for this comment, which aligns with Question 3 raised by Reviewer 1. In response, we have added a dedicated section discussing the relevance of novel CAR designs in addressing this important aspect (lines 237-241).

**Target expression is a very big topic for CAR-T cell development, but also for antibody development. Please also make a comparison to what antigen expression is required for antibody treatments.**

We thank the reviewer for this comment. While we acknowledge that this topic may have implications for antibody-based treatments, we believe that a direct comparison is less feasible due to the differences in the mechanisms of action of these medicinal products.
CAR T-cell function is independent of natural signaling and interactions with other immune cells. In contrast, the function of antibody-based therapies is dependent on and can be limited by factors beyond the characteristics of the CD19-expressing target cell. For instance, monoclonal antibodies like tafasitamab require the triggering of immune effector functions, such as antibody-dependent cellular cytotoxicity, as well as an active host immune system. The activity of T-cell engagers (e.g., blinatumomab) is influenced by the specific affinities of both the CD19 and CD3 binding domains, and is heavily dependent on the functional integrity of the engaged T cells. Lastly, antibody-drug conjugates like loncastuximab tesirine combine an anti-CD19 antibody with a cytotoxic payload, and their efficacy relies not only on CD19 binding but also on the internalization and intracellular action of the payload. Furthermore, in a context of heterogenous target expression within the tumor, use of cell-permeable payloads released from the ADC exploits the mechanism of “bystander killing”, enabling even eradication of target-low to -negative tumor cells.
Therefore, we believe that a direct comparison of CD19 expression thresholds required for the efficacy of these different therapies is not feasible. We hope the reviewer will consider and accept this perspective.
While writing this reply and reconsidering the topic, we also decided to remove the reference on the theoretical calculated loncastuximab tesirine affinity (line 176).

14th Apr 2026

Dear Dr. Sebe,

Thank you for submitting your revised manuscript to EMM. Referee #2 has now reviewed the revision and is satisfied with the changes overall, but has raised one remaining minor comment that we would like you to address. Aside from this point, everything is in order, and I will be pleased to accept your manuscript once it has been resolved.

Looking forward to receiving your revised manuscript at your earliest convenience.

With kind regards,

Lise Roth

Lise Roth, Ph.D
Senior Editor
EMBO Molecular Medicine

***** Reviewer's comments *****

Referee #2 (Remarks for Author):

The authors have addressed my concerns. This is an important and highly topical article.

I have one minor point to be discussed. While I understand the author´s reply on the difference between CAR T cells and antibody with respect to the target antigen expression, I still believe that the article should discuss what "target expression" means in the context of CAR T cells versus antibody treatments.
It is a question that commonly concerns clinicians, whether a patient not responding to antibodies may respond to subsequent treatment with CAR T cells against the same target, or whether patients relapsing after CAR T treatment may respond to antibody treatment against the same target (if tumor cells are positive).

The authors addressed the remaining editorial issues.

21st Apr 2026

Dear Dr. Sebe,

Thank you for submitting your revised manuscript. I am pleased to inform you that your manuscript is accepted for publication and is now being sent to our publisher to be included in the next available issue of EMBO Molecular Medicine.

Your manuscript will be processed for publication by EMBO Press. It will be copy edited and you will receive page proofs prior to publication. Please note that you will be contacted by Springer Nature Author Services to complete licensing information. This Perspective article is free of charge.

If you have any questions, please do not hesitate to contact the Editorial Office. Thank you for your contribution to EMBO Molecular Medicine.

With kind regards,

Lise

Lise Roth, Ph.D
Senior Editor
EMBO Molecular Medicine

------------------------------------------------
